# Design, Synthesis and DNA Interaction Study of New Potential DNA Bis-Intercalators Based on Glucuronic Acid

**DOI:** 10.3390/ijms140816851

**Published:** 2013-08-15

**Authors:** Jiuyang Zhao, Wei Li, Rui Ma, Shaopeng Chen, Sumei Ren, Tao Jiang

**Affiliations:** 1 Key Laboratory of Marine Drugs, The Ministry of Education of China, School of Pharmacy, Ocean University of China, Qingdao 266003, China; E-Mails: flyawayzhao@163.com (J.Z.); lwlw1919@163.com (W.L.); xrzhsh_2006@126.com (R.M.); chenshaopeng2006@163.com (S.C.); rensumei@ouc.edu.cn (S.R.); 2 School of Pharmacy, Jining Medical University, Rizhao 276826, China

**Keywords:** glycuronic acid, DNA bis-intercaltor, synthesis and design

## Abstract

A series of novel potential DNA bis-intercalators were designed and synthesized, in which two glucuronic acids were linked by ethylenediamine, and the glucuronic acid was coupled with various chromophores, including quinoline, acridine, indole and purine, at the C-1 position. The preliminary binding properties of these compounds to calf thymus DNA (CT-DNA) have been investigated by UV-absorption and fluorescence spectroscopy. The results indicated that all the target compounds can interact with CT-DNA, and the acridine derivative, **3b**, showed the highest key selection vector (KSV) value, which suggested that compound **3b** binds most strongly to CT-DNA.

## 1. Introduction

Numerous biological experiments have suggested that DNA is one of the primary cellular targets for many anticancer agents. Particularly, in cancer cells, DNA can be preferentially damaged, due to the interactions with anticancer agents, therefore inhibition/blockage of cell division causes cell death [1–5]. The DNA interacting molecules are usually bound to DNA non-covalently by three modes: intercalation, groove binding and static electronic interactions. Static electronic interactions refers to molecules that bind with the negatively charged DNA double helix externally through a non-specific interaction. In groove binding, the targeting molecules interact with DNA in the base edges of the major groove or minor groove, which had been discussed by many groups [6–10]. The intercalation is another DNA binding mode that is closely related to the antitumor ability of many anticancer agents [11].

Intercalators are a group of compounds that interact with the DNA double helix in a reversible manner. Some of them are valuable drugs currently used for the treatment of ovarian and breast cancers, as well as acute leukemia, while many others are in different phases of clinical trials. Intercalating agents share some common structural features, such as the presence of planar polyaromatic systems that are inserted between DNA base-pairs with a marked preference for 5′-pyrimidine-purine-3′ sequence. In addition, the chromophores are linked to basic moieties that might also play an important role in the affinity and selectivity shown for compounds [12–15].

Bis-intercalators have two potential intercalating ring systems connected by linkers that can vary in both length and rigidity. In the 1990s, Priebe and co-workers developed the bis-intercalators of daunomycin, which had expanded research ideas [16]. In recent years, the synthesis of bis-intercalators has drawn considerable attention, because, in comparison with mono-intercalators, higher DNA-binding constants, slower dissociation rates and substantial sequence selectivity can be expected by incorporating two or more intercalating units into a polyfunctional ligand [17]. In addition, the interactions with the groove or phosphate groups by the linker may provide enhancement for binding affinity or selectivity [18].

**Figure 1:**
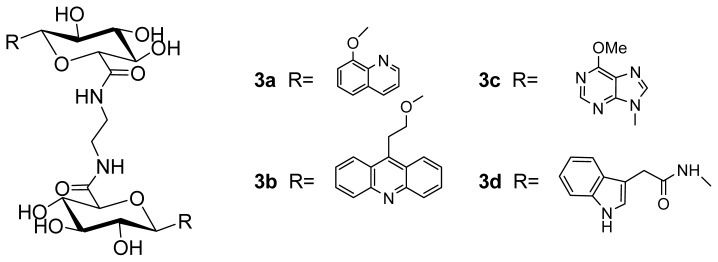
DNA bis-intercalators **3a**–**3d**.

A number of sugar-intercalator conjugates have been studied before for different nucleic acid binding [19–24], and monosaccharides have been introduced as biologically relevant scaffolds. The advantage of using saccharides is to acquire the high density of functional groups. Meanwhile, they are available as single enantiomers and contain multiple sites for attachment of recognition groups [25,26]. In this work, we report the design and synthesis of four potential DNA bis-intercalators based on glucuronic acid (Figure 1). Glucuronic acids, which have a 6-COOH rather than a 6-CH_2_OH, are in glycosides converted to amides via coupling with an amine. The intercalation binding force comes from the π–π interactions and hydrophobic interactions between the aromatic ring of the intercalator and the DNA base, so we chose the planar aromatic molecules, quinoline, acridine, indole and purine, as chromophores to incorporate into these bis-intercalators. The interactions of these bis-intercalators with calf thymus DNA (CT-DNA) were studied.

## 2. Results and Discussion

### 2.1. Synthesis

The starting material, glucurono-3,6-lactone, was converted into methyl tetra acetyl glucopyranuronate **2** by methyl esterification and acetylation, according to the literature method [27]. The glycosylating agents, such as glucuronosyl bromide **3**, glucuronosyl azide **4** and glucuronosylamine **5**, were prepared by bromization, azide substitution and hydrogenation in good yield based on methods described in the literature [28–30].

**Scheme 1:**
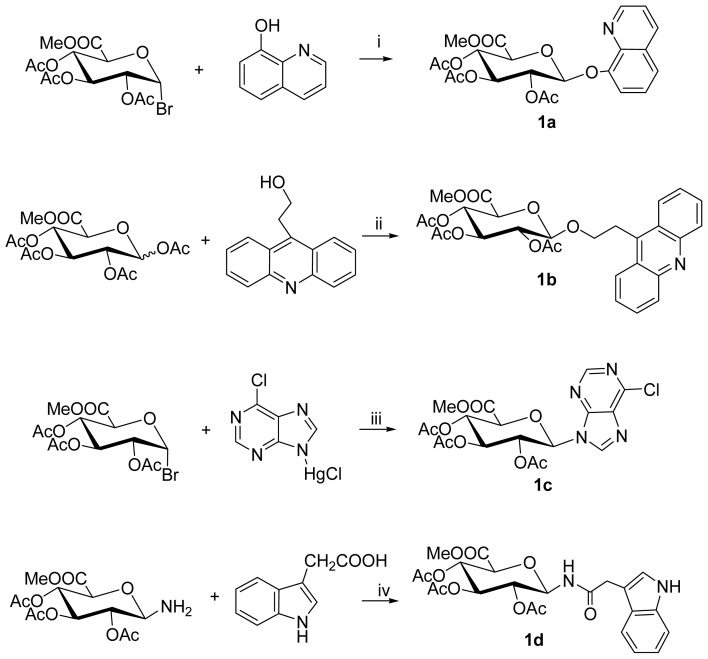
Reagents and conditions: (**i**) Ag_2_O, CH_2_Cl_2_, 58%; (**ii**) Trimethylsilyl trifluoromethanesulfonate (TMSOTf), CH_2_Cl_2_, 35%; (**iii**) CdCO_3_, Celite, toluene, reflux; (**iv**) 1-ethyl-3-(3-dimethylaminopropyl)carbodiimide (EDCI), tetrahydrofuran (THF), 30%.

Then, the acetyl protected glucuronosyl donor was coupled with the derivatives of quinoline, acridine, purine and indole, respectively, to give the methyl (aryl glucuronosyl)-uronates. Compound **1a** was synthesized (57.6% yield) via the reaction of **3** with 8-hydroxylquinoline (3 Eq.) using Ag_2_O (5 Eq.) as catalyst. Trimethylsilyl trifluoromethanesulfonate (TMSOTf) promoted coupling of **2**, with 9-β-hydroxylethyl acridine giving **1b** (24.2% yield). The reaction of **3** with Cloromercuri-6-chloropurine catalyzed by CdCO_3_ gave **1c** (35% yield) [31]. Compound **1d** was prepared from the azide **4** through reduction to the amine **5**, and subsequent coupling was performed with 2-(indol-3-yl)acetic acid promoted by 1-ethyl-3-(3-dimethylaminopropyl)carbodiimide (EDCI) [30]. (Scheme 1).

**Scheme 2:**
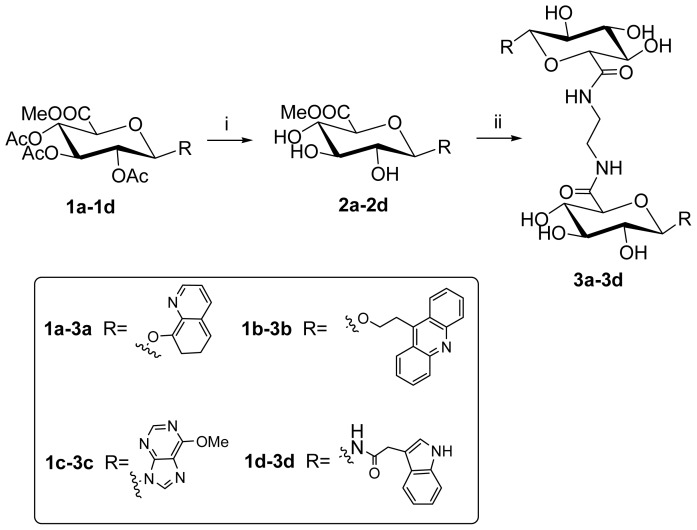
Reagents and conditions: (**i**) MeONa, MeOH; (**ii**) H_2_N (CH_2_)_2_NH_2_, MeOH, reflux, yield 15%~25%.

Deacetylation by MeONa/MeOH and treatment of these (aryl glucuronosyl)-uronates (**2a**–**2d**) with 0.5 eq. ethylenediamine in methanol under reflux afforded the target bis-intercalators (compound **3a**–**3d**, yield 15%~25% Scheme 2).

### 2.2. DNA Binding Properties

To study how efficiently the synthesized compounds interact with CT-DNA, we investigated their DNA binding primary properties by UV absorption and fluorescence emission spectra.

#### 2.2.1. UV Absorption

The measurement of UV absorption of compounds was conducted in the phosphate buffer by using a fixed compound concentration (2 × 10^−5^ M) to which increments of the CT-DNA stock solution was added. The solutions were allowed to incubate for 5 min before the absorption spectra were recorded. The UV absorption band of compound **3c** and **3d** was similar to that of the DNA duplex, around the wavelength of 260 nm; only UV absorption spectra of compound **3a** and **3b** were measured.

**Figure 2:**
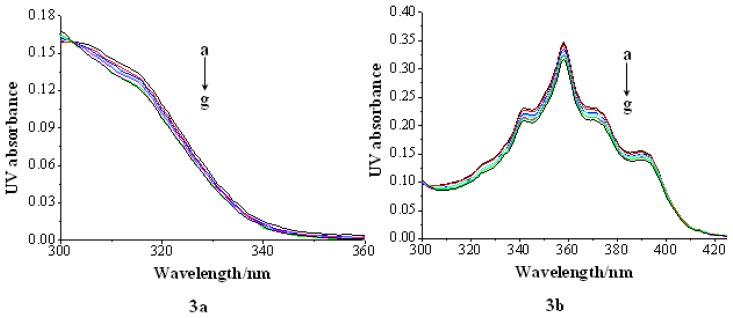
Absorption spectra of compound **3a**, **3b** (2 × 10^−5^ mol/L) in the presence of calf thymus DNA (CT-DNA) in phosphate buffer containing. [DNA]: (**a**) 0; (**b**) 2 × 10^−5^ mol/L; (**c**) 4 × 10^−5^ mol/L; (**d**) 6 × 10^−5^ mol/L; (**e**) 8 × 10^−5^ mol/L; (**f**) 1 × 10^−4^ mol/L; (**g**) 1.2 × 10^−4^ mol/L.

The change in compound **3a** and **3b** UV absorption spectra with increasing concentration of CT-DNA is shown in Figure 2. Specifically, both compounds showed hypochromicity upon increasing DNA concentration, indicating that both compounds interacted with the DNA helix. However, a weak hypochromic effect is indeed observed, and no red shift is apparent. So, these changes were not significant enough to accurately determine the DNA-binding constants.

#### 2.2.2. Fluorescence Emission

**Figure 3:**
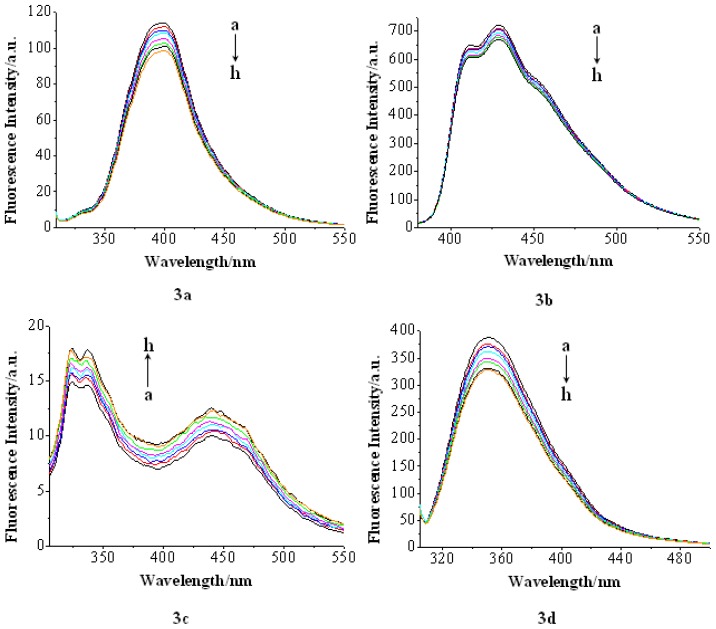
Fluorescence spectra of **3a**–**3d** (2 × 10^−5^ mol/L) in the presence of CT-DNA in pH = 7.0 phosphate buffer containing. [DNA]: (**a**) 0; (**b**) 1 × 10^−5^ mol/L; (**c**) 2 × 10^−5^ mol/L; (**d**) 3 × 10^−5^ mol/L; (**e**) 4 × 10^−5^ mol/L; (**f**) 5 × 10^−5^ mol/L; (**g**) 6 × 10^−5^ mol/L; (**h**) 7 × 10^−5^ mol/L. Arrows show the intensity change upon increasing DNA concentrations. **3a**: λ_ex_ = 300 nm, **3b**: λ_ex_ = 350 nm, **3c**: λ_ex_ = 290 nm, **3d**: λ_ex_ = 300 nm.

The fluorescence titration experiment has been widely used to characterize the compound-DNA interactions, in which the fluorescence emissions of interacting compounds can be quenched, which results in the decrease of fluorescence intensity [32,33]. On the other hand, in some compounds, the compound-DNA interactions can prevent the compound fluorescence emission from being quenched by polar solvent molecules. Consequently, the fluorescence intensity increases [33]. In this study, the interactions between the compounds and CT-DNA were investigated by fluorescence titration. The results are shown in Figure 3. Specifically, the fluorescence intensity of compound **3a**, **3b** and **3d** decreased when titrated by CT-DNA, being in good agreement with the fluorescence behavior of other intercalators reported in the literature [17]. On the contrary, an increase in the fluorescence intensity of compound **3c** was observed. The results indicated that all the compounds can interact with CT-DNA, whereas the binding mode may be different.

#### 2.2.3. EB Competition Assay

The well-established quenching assay based on the displacement of the intercalating dye, ethidium bromide (EB), from CT-DNA was employed to further investigate the interaction mode between the complexes and CT-DNA. EB is a very useful DNA structural probe, which shows a significant increase in fluorescence intensity when intercalating into the base pair of DNA. However, the enhanced fluorescence can be quenched evidently when there is a second complex that can replace the bound EB or break the secondary structure of DNA [33–35]. It has been reported that the groove DNA binders can also cause the decrease in EB emission intensities. The effects were, however, only moderate [36].

Before we started the fluorescence test of the compounds with DNA, we tried different excitation wavelengths from 280 to 550 nm and found only one fluoresce for each compound, and each had a fixed wavelength, so we chose the excitation wavelength that is similar with the UV absorbance one. All the compounds do not fluoresce with an excitation at 518 nm.

**Figure 4:**
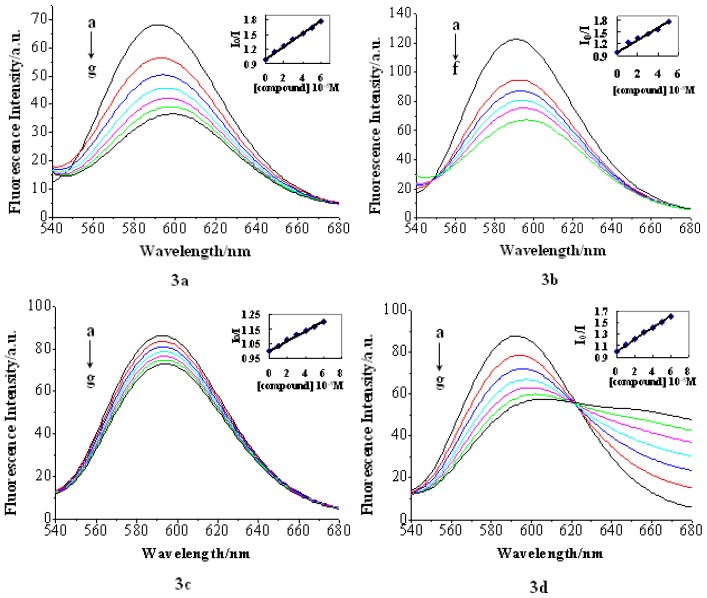
Fluorescence changes that occur when the CT-DNA-ethidium bromide (EB) system is titrated with **3a**–**3d** in phosphate buffer (pH = 7.0), λ_ex_ = 518 nm, [EB] = 1 × 10^−5^ mol/L, [DNA] = 1 × 10^−4^ mol/L; [compound]: (**a**) 0; (**b**) 1 × 10^−5^ mol/L; (**c**) 2 × 10^−5^ mol/L; (**d**) 3 × 10^−5^ mol/L; (**e**) 4 × 10^−5^ mol/L; (**f**) 5 × 10^−4^ mol/L; (**g**) 6 × 10^−5^ mol/L. Arrows show the intensity change upon increasing DNA concentrations. Inset: plot of I_0_/I *vs.* [compound] for the titration of the compound to CT-DNA-EB.

The EB competition assay results are shown in Figure 4. The fluorescence intensity of DNA-bound EB at 586 nm decreased remarkably with the increase of the compound (**3a**–**3d**) concentrations. This decrease in fluorescence intensity may be due to the quenching of some EB molecules that were released from DNA into the solution after being replaced by the compounds.

The similar phenomenon that the fluorescence of DNA-bound EB was quenched as a result of the DNA and compound interactions, a characteristic sign of intercalation [37], has also been reported for the chitosan complex [38].

The fluorescence quenching of DNA-bound EB can be well described by the linear Stern-Volmer equation [39] in which the synthesized compounds were the quenchers:

(1) $$I_{0}/I=1+KSV\;[Q]$$

*I*_0_ and *I* represent the fluorescence intensities in the absence and presence of quencher, respectively; *KSV* is a linear Stern-Volmer quenching constant; *Q* is the concentration of quencher. The KSV values were given by the ratio of the slope to intercept. The KSV values for the tested compounds are listed in Table 1.

As shown is Table 1, compound **3b** had the highest KSV value, which suggested that compound **3b** bound most strongly to CT-DNA. The classic intercalators, such as acridine derivatives, seemed to be the most efficient. Further investigations are being currently conducted in our laboratory to obtain an efficient dye group (or proper linker) for the bis-intercalators.

## 3. Experimental Section

### 3.1. General Methods

All reagents and solvents were purchased commercially and purified in a conventional manner. Thin layer chromatography (TLC) was performed on precoated E. Merck Silica Gel 60 F254 plates. Flash column chromatography was performed on silica gel (200–300 mesh, Qingdao, China). ^1^H NMR and ^13^C NMR spectra were taken on a Jeol JNM-ECP 600 spectrometer with tetramethylsilane (Me_4_Si) as the internal standard, and chemical shifts are recorded in d-values. Mass spectra were recorded on a Q-TOF Global mass spectrometer.

### 3.2. DNA-Binding Studies

Calf thymus DNA (CT-DNA) was obtained from Sigma (Sigma, St. Louis, MO, USA). All experiments involving CT-DNA were performed in 10 mM phosphate buffered saline (PBS, 100 mM NaCl, pH = 7.0). The per nucleotide DNA concentration was determined by absorption at 260 nm [40] with the molar absorption coefficient of 6600 M^−1^ cm^−1^. A ratio of 260 nm/280 nm around 1.8–1.9 indicated high quality of the CT-DNA sample (free of protein contamination) [41]. Stock DNA solutions (*c* = 1.67 × 10^−2^ M) were stored at 4 °C and for no longer than 4 days before use (1.8 μL solution was added to the cuvette, which makes the concentration of DNA 1 × 10^−5^ M, and the added total volume is 10.8 μL when the ratio of compound to DNA is 1:3).

The UV-visible absorption spectra of DNA binding studies were performed on a Shimadzu UV-2550 spectrophotometer (Shimadzu Co., Kyoto, Japan). The fluorescence spectra were measured with a Fp-750w Fluorometer. The absorption and fluorescence titration samples contained small aliquots of DNA solution and the same concentration of the synthesized compound. All the tested compounds were evaluated in 50% DMSO, because they were poorly soluble. The fluorescence spectra of the complexes were recorded by using the excitation wavelength of 300 nm for **3a** and **3d**, 350 nm for **3b** and 290 nm for **3c**. The emission wavelength were as follows: 399 nm for **3a**, 429 nm for **3b**, 324 nm for **3c** and 350 nm for **3d**. Before measurements, the mixture was mixed well and incubated at room temperature for 5 min.

In the ethidium bromide (EB) fluorescence displacement experiment, 5 μL of the EB Tris solution (1 mM) was added to 1 mL of DNA solution (1 × 10^−4^ mol/L, at saturated binding level) [42], followed by a 2 h incubation in the dark. The DNA/compound complex was then titrated into the EB/DNA mixture. Before measurements, the solution was well mixed and incubated at room temperature for 30 min. Fluorescence spectra of EB bound to DNA were obtained at an excitation wavelength of 518 nm and an emission wavelength of 586 nm.

### 3.3. Synthesis

#### 8-*O*-(methyl 2,3,4-tri-*O*-acetyl-β-d-glucuronosyluronate)quinolin (**1a**)

To a solution of **2** (1.0 g, 2.5 mmol) and 8-hydroxylquinolin (1.08 g, 7.5 mmol) in dry CH_2_Cl_2_, (25 mL) was added freshly prepared Ag_2_O (2.9 g, 12.5 mmol). The resulting solution was allowed to stir 12 h at room temperature (r.t.) away from light, then diluted with EtOAc (25 mL) and filtered through Celite. The filtrate was washed with aqueous saturated NaHCO_3_ and water, followed by drying over MgSO_4_. The resulting solution was further concentrated under reduced pressure and purified by silica gel chromatography (petroleum ether: EtOAc = 3:2) to yield product **1a** (0.64 g, 57.6%) (white solid, mp 66–68 °C). ^1^H NMR(CDCl_3_, 600 MHz): δ: 8.90 (dd, 1H, *J* = 1.4, 4.1 Hz, H-2), 8.14 (dd, 1H, *J* = 1.9, 8.2 Hz, H-4), 7.59 (dd, 1H, *J* = 2.8, 6.4 Hz, H-5), 7.46 (m, 2H, H-3, H-6), 7.43 (dd, 1H, *J* = 4.1, 8.3 Hz, H-7), 5.57 (d, 1H, *J* = 7.8 Hz, H-1′), 5.43 (m, 3H, H-3′, H-4′,H-5′), 4.16 (dd, 1H, *J* = 2.3, 6.8 Hz, H-2′), 3.7 (s, 3H, OC*H*_3_), 2.04–2.10 (3s, each 3H, Ac-H); ESI-MS: *m*/*z* [M+H]^+^: 462.9.

#### 8-*O*-(methyl β-d-glucuronosyluronate)quinolin (**2a**)

A solution of **1a** (0.6 g, 1.3 mmol) in methanol (15 mL) was treated with sodium methoxide (0.4 mol/L, 0.6 mL) for 6 h at room temperature. The mixture was acidified to pH 6 by addition of Amberlite IR-120 (H^+^). The resin was removed by filtration, the filtrate was concentrated and a white solid (**2a**, 0.32 g, 73.5% yield) was obtained. mp 133–135 °C. ^1^H NMR(CDCl_3_, 600 MHz) δ: 8.89 (dd, 1H, *J* = 1.8, 4.1 Hz, H-2), 8.37 (dd, 1H, *J* = 1.9, 8.3 Hz, H-4), 7.64 (d, 1H, *J* = 7.8 Hz, H-5), 7.58 (dd, 1H, *J* = 4.1, 8.3 Hz, H-3), 7.54 (dd, 1H, *J* = 8.2, 7.8 Hz, H-6), 7.40 (dd, 1H, *J* = 0.9, 7.8 Hz, H-7), 5.38 (d, 1H, *J* = 7.8 Hz, H-1′), 4.12 (d, 1H, *J* = 9.7 Hz, H-2′), 3.65 (s, 3H, OCH_3_), 3.38–3.51 (m, 3H, H-3′, H-4′, H-5′); ESI-MS: *m*/*z* [M+H]^+^: 336.2.

#### *N*,*N*-1,2-di-(1-*O*-(quinolin-8-yl)-β-d-glucuronamide)ethane (**3a**)

Ethylenediamine (0.03 g, 0.5 mmol) was added to a solution of **1a** (0.32 g, 0.95 mmol) in methanol (15 mL). The reaction mixture was stirred under reflux for 2 days, then cooled to room temperature, and the precipitate formed was filtered and washed with hot methanol (10 mL × 3). The titled compound, **3a** (80 mg, 25.0% yield), was obtained as a white solid. mp 202–205 °C. ^1^H NMR (DMSO-*d*_6_, 600 MHz) *δ*: 8.89 (dd, 1H, *J* = 1.4, 4.1 Hz, H-2), 8.37 (dd, 1H, *J* = 1.9, 8.3 Hz, H-4), 8.08 (s, 1H, NH), 7.64 (d, 1H, *J* = 7.3 Hz, H-5), 7.58 (dd, 1H, *J* = 4.1, 8.3 Hz, H-3), 7.54 (t, 1H, *J* = 7.8, 8.3 Hz, H-6), 7.41 (d, 1H, *J* = 7.8 Hz, H-7), 5.23–5.77 (3d, 3H, 3OH), 5.20 (d, 1H, *J* = 7.7 Hz, H-1′), 3.81 (d, 1H, *J* = 9.6 Hz, H-2′), 3.48 (m, 2H, H-4′, H-5′), 3.12 (b, 2H, CH_2_); ^13^C NMR (DMSO-*d*_6_, 150 MHz) δ: 168.45 (C=O), 152.3, 149.3, 139.7, 136.2, 129.1, 126.8, 121.9, 121.8, 114.1, 101.0, 76.2, 75.9, 73.1, 71.2, 38.1; ESI-MS: *m*/*z* [M+H]^+^: 667.2; HRMS(ESI): calcd. for C_32_H_35_N_4_O_12_^+^ 667.2251, found 667.2276.

#### 9-*O*-(methyl 2,3,4-tri-*O*-acetyl-β-d-glucuronosyluronate)ethyl acridine (**1b**)

To a solution of methyl tetra acetyl-β-d-glucopyranuronate (1.7 g, 4.5 mmol) and 9-β-hydroxyethyl-acridine (0.94 g, 4.2 mmol) in dry CH_2_Cl_2_ (25 mL) was added dropwise to a solution of TMSOTf (1.13 mL, 6.75 mmol) in CH_2_Cl_2_ (10 mL) on an ice bath. The reaction mixture was allowed to stir on the ice bath for 30 min, and then it was removed and allowed to stir at r.t. overnight. The reaction mixture was diluted with further CH_2_Cl_2_ (30 mL), then washed with aqueous saturated NaHCO_3_ and water, dried over MgSO_4_. After filtration the solvent was removed under reduced pressure to give a mixture of products that was purified by column chromatography (petroleum ether: EtOAc = 2:1) to give 0.55 g (24.2% yield) of **1b** as a light yellow solid (mp 106–110 °C). ^1^H NMR (DMSO-*d*_6_, 600 MHz) δ: 8.39 (m, 2H, H-4, H-5), 8.14 (m, 2H, H-1, H-8), 7.84 (dd, 2H, *J* = 7.3, 7.8 Hz, H-3, H-6), 7.61 (dd, 2H, *J* = 7.7, 7.4 Hz, H-2, H-7), 5.23 (d, 1H, *J* = 9.6 Hz, H-1′), 4.92 (dd, 1H, *J* = 10.1, 9.6 Hz, H-4′), 4.89 (t, 1H, *J* = 7.7 Hz, H-3′), 4.69 (dd, 1H, *J* = 9.6, 8.3 Hz, H-2′), 4.43 (d, 1H, *J* = 10.1 Hz, H-5′), 4.01–3.93 (m, 4H, CH_2_CH_2_), 3.64 (s, 3H, OCH_3_), 1.96 (s, 3H, Ac-H), 1.90 (s, 3H, Ac-H), 1.58 (s, 3H, Ac-H); ESI-MS: *m*/*z* [M+H]^+^: 540.1.

#### 9-*O*-(methyl β-d-glucuronosyluronate)ethyl acridine (**2b**)

To a solution of **1b** (0.54 g, 1.0 mmol) in methanol (15 mL) was treated with sodium methoxide (0.4 mol/L, 0.8 mL) for 12 h at room temperature. The reaction mixture was acidified to pH 6 by addition of Amberlite IR-120 (H^+^). The resin was removed by filtration, and the filtrate was concentrated, then purified on silica gel column chromatography eluted with EtOAc to give 0.26 g (63.4% yield) of **2b** as a light yellow solid (mp 143–145 °C). ^1^H NMR (DMSO-*d*_6_, 600 MHz) *δ*: 8.44 (m, 2H, H-4, H-5), 8.15 (m, 2H, H-1, H-8), 7.85 (m, 2H, H-3, H-6), 7.64 (m, 2H, H-2, H-7), 5.34 (d, 1H, *J* = 5.9 Hz), 5.17 (d, 1H, *J* = 5.0 Hz), 5.16 (d, 1H, *J* = 5.0 Hz), 4.47 (d, 1H, *J* = 7.8 Hz, H-1′), 4.04–3.92 (m, 4H, CH_2_CH_2_), 3.82 (d, 1H, *J* = 9.7 Hz, H-2′), 3.67 (s, 3H, OCH_3_), 3.21 (m, 1H, H-5′), 3.02 (m, 1H, H-3′); ESI-MS: *m*/*z* [M+H]^+^: 414.2.

#### *N*,*N*-1,2-di-(1-*O*-(2-(acridine-9-yl)ethyl)-β-d-glucuronamide) ethane (**3b**)

This compound was prepared from **2b** (0.24 g, 0.58 mmol) and ethylenediamine (0.017 g, 0.29 mmol) using the same procedure as described for **3a**. Purification of the residue resulted in **3c** (40 mg, 16.7% yield) as a light yellow solid. mp 213–216 °C. ^1^H NMR (DMSO-*d*_6_, 600 MHz) *δ*: 8.42 (m, 2H, H-4, H-5), 8.12 (m, 2H, H-1, H-8), 8.03 (s, 1H, NH), 7.81 (t, 2H, *J* = 7.3, 7.8 Hz, H-3, H-6), 7.62 (t, 2H, *J* = 7.7, 7.4 Hz, H-2, H-7), 5.10 (m, 3H, 3OH), 4.35 (d, 1H, *J* = 7.8 Hz, H-1′), 3.94 (m, 4H, CH_2_CH_2_), 3.57 (d, 1H, *J* = 9.6 Hz, H-2′), 3.17 (m, 1H, H-5′), 3.15 (b, 2H, CH_2_N), 3.04 (m, 1H, H-3′). ^13^C NMR (DMSO-*d*_6_, 150 MHz) *δ*: 168.9, 148.0, 142.50, 123.0, 129.7, 125.9, 124.9, 124.9, 103.4, 76.1, 75.7, 73.1, 71.4, 69.0, 38.2, 28.0; ESI-MS: *m*/*z* [M+H]^+^: 823.6; HRMS (ESI): calcd. for C_44_H_46_N_4_O_12_Na^+^ 845.3010, found 845.3045.

#### 6-Cloro-9-(methyl 2,3,4-tri-*O*-acetyl-1-deoxy-β-d-glucuronosyluronate)purine (**1c**)

Glucuronosyl bromide **3** (3.0 g, 7.5 mmol) was added to an azeotropically dried mixture of Cloromercuri-6-chloropurine (3.5 g, 9 mmol), Celite (3 g) and cadmium (3.3 g, 18.75 mmol) in toluene (150 mL), followed by 3 h of heating under reflux and then filtrated through Celite. The hot CH_2_Cl_2_-soluble material was collected from the filter cake and from the residue obtained on evaporation of the filtrate. The extract was washed twice with aqueous potassium iodide, twice with water and dried over MgSO_4_ and concentrated. The residue was purified on silica gel column chromatography (petroleum ether: EtOAc = 3:2) to give 1.25 g (35% yield) of **1c** as a white solid (mp 223–224 °C). ^1^H NMR (DMSO-*d*_6_, 600 MHz) *δ*: 9.12 (s, 1H, H-2), 8.88 (s, 1H, H-8), 6.45 (d, 1H, *J* = 9.2 Hz, H-1′), 5.98 (dd, 1H, *J* = 9.2, 9.6 Hz, H-3′), 5.75 (dd, 1H, *J* = 9.6, 9.2 Hz, H-2′), 5.32 (dd, 1H, *J* = 9.6, 10.1 Hz, H-4′), 4.92 (d, 1H, *J* = 10.1 Hz, H-5′), 3.62 (s, 3H, OCH_3_), 1.71–2.04 (3s, each3H, Ac-H); ^13^C NMR (DMSO-*d*_6_, 150 MHz); *δ*: 169.5, 169.4, 168.9, 152.4, 151.7, 149.6, 145.8, 130.7, 79.3, 72.8, 71.2, 69.8, 68.4, 52.6, 20.3, 19.9; ESI-MS: *m*/*z* [M+H]^+^: 471.0.

#### 6-methoxyl-9-(methyl β-d-glucuronosyluronate)purine (**2c**)

A solution of **1c** (1.2 g, 2.55 mmol) in methanol (35 mL) was treated with sodium methoxide (0.4 mol/L, 8.75 mL, 3.5 mmol) for 8 h at room temperature. The reaction mixture was acidified to pH 6 by addition of Amberlite IR-120 (H^+^). The resin was removed by filtration, and the filtrate was concentrated, then purified on silica gel column chromatography eluted with EtOAc to give 0.66 g (76.7% yield) of **2c** as a white solid. mp 245–247 °C. ^1^H NMR (DMSO-*d*_6_, 600 MHz) *δ*: 8.61 (s, 1H, H-2), 8.57 (s, 1H, H-8), 5.66 (d, 1H, *J* = 9.2 Hz, H-1′), 5.56 (d, 1H, *J* = 6.0 Hz, OH), 5.53 (d, 2H, *J* = 5.5 Hz, 2OH), 4.19 (m, 1H, H-2′), 4.12 (d, 1H, *J* = 9.6 Hz, H-5′), 4.10 (s, 1H, OCH_3_), 3.64 (s, 1H, COOC*H*_3_), 3.57 (m, 1H, H-4′), 3.48 (m, 1H, H-3′); ^13^C NMR (DMSO-*d*_6_, 150 MHz) δ: 168.7, 160.34, 152.2, 151.9, 142.7, 120.7, 83.1, 77.6, 76.2, 71.2, 70.5, 54.0, 52.0; ESI-MS: *m*/*z* [M+H]^+^: 341.0.

#### *N*,*N*-1,2-di-(1-deoxy-1-(6-chloropurine-9-yl)-β-d-glucuronamide)ethane (**3c**)

This compound was prepared from **2c** (0.63 g, 1.85 mmol) and ethylenediamine (0.06 g, 0.97 mmol) using the same procedure as described for **3a**. Purification of the residue with silica gel column chromatography (EtOAc: MeOH = 3:1) resulted in **3c** (120 mg, 19.3% yield) as a white solid. mp > 250 °C. ^1^H NMR (DMSO-*d*_6_, 600MHz) δ: 8.62 (s, 1H, H-2), 8.56 (s, 1H, H-8), 8.16 (br s, 1H, NH), 5.56 (d, 1H, *J* = 9.2 Hz, H-1′), 5.46 (d, 1H, *J* = 5.9 Hz, OH), 5.45 (d, 1H, *J* = 4.6 Hz, OH), 5.37 (d, 1H, *J* = 5.0 Hz, OH), 4.11 (s, 3H, OCH_3_), 4.06 (m, 1H, H-2′), 3.85 (d, 1H, *J* = 9.6 Hz, H-5′), 3.57 (m, 1H, H-4′), 3.43 (m, 1H, H-3′), 3.08 (br s, 2H, NCH_2_); ^13^C NMR (DMSO-*d*_6_, 150 MHz) *δ*: 167.9, 160.3, 152.2, 151.8, 142.5, 120.6, 82.9, 78.1, 76.6, 71.0, 70.9, 54.0, 38.0; ESI-MS: *m*/*z* [M+H]^+^: 676.3; HRMS (ESI): calcd. for C_26_H_33_N_10_O_12_^+^ 677.2279, found 677.2290.

#### *N*-(methyl-2,3,4-tri-*O*-acetyl-1-deoxy-β-d-glucuronosyluronate)-2-(indol-3-yl) acetamide (**1d**)

Reaction of azide **4** with Pd/C in dry THF at −5 °C under H_2_ gave amine **5** and its α-anomer in a 13:1 ratio after filtration and removal of solvent. The freshly prepared amine was added to a solution of 2-(indole-3-yl) acetic acid (1.1 g, 6 mmol) and EDCI (1.15 g, 6 mmol) in 50 mL THF containing 0.5 mL water. The reaction mixture was stirred for 24 h at room temperature. After being concentrated, the residue was dissolved in 50 mL EtOAc and washed three times with water. The organic phase was dried over MgSO_4_ and filtered, and the solvent was removed under reduced pressure to give a mixture of products that was purified by column chromatography (petroleum ether: EtOAc = 1:1) to give 0.87 g (29.6% yield) of **1d** as a white solid (mp 198–200 °C). ^1^H NMR (DMSO-*d*_6_, 600 MHz) δ: 10.90 (s, 1H, indol-NH), 8.84 (d, 1H, *J* = 9.7 Hz, sugar-NH), 7.48 (d, 1H, *J* = 7.8 Hz, H-4), 7.33 (d, 1H, *J* = 7.7 Hz, H-7), 7.15 (d, 1H, *J* = 2.3 Hz, H-2), 7.05 (m, 1H, H-5), 6.94 (m, 1H, H-6), 5.47 (dd, 1H, *J* = 9.2, 9.6 Hz, H-1′), 5.41 (dd, 1H, *J* = 9.6, 9.7 Hz, H-3′), 4.90 (m, 2H, H-4′, H-5′), 4.56 (d, 1H, *J* = 9.6 Hz, H-2′), 3.62 (s, 3H, OCH_3_), 3.52 (d, 2H, indol-CH_2_), 1.98 (s, 3H, Ac-H), 1.93 (s, 3H, Ac-H), 1.67 (s, 3H, Ac-H); ESI-MS: *m*/*z* [M+H]^+^: 491.1.

#### *N*-(methyl β-d-glucuronosyluronate)-2-(indol-3-yl)acetamide (**2d**)

A solution of **1d** (0.83 g, 1.7 mmol) in methanol (25 mL) was treated with methanolic NaOMe (0.4 mol/L, 0.8 mL) for 8 h under ice bath. The reaction mixture was acidified to pH 6 by addition of Amberlite IR-120 (H^+^). The resin was removed by filtration, the filtrate was concentrated and a brown solid (**2d**, 0.6 g, 97.4% yield) was obtained (mp 186–188 °C). ^1^H NMR (DMSO-*d*_6_, 600MHz) δ: 10.86 (s, 1H, indole-NH), 8.66 (d, 1H, *J* = 9.2 Hz, sugar-NH), 7.55 (d, 1H, *J* = 7.8 Hz, H-4), 7.32 (d, 1H, *J* = 8.3 Hz, H-7), 7.19 (d, 1H, *J* = 2.7 Hz, H-2), 7.05 (m, 1H, H-5), 6.95 (m, 1H, H-6), 5.30 (d, 1H, *J* = 5.9 Hz, OH), 5.19 (d, 1H, *J* = 5.0 Hz, OH), 5.06 (d, 1H, *J* = 5.5 Hz, OH), 4.80 (dd, 1H, *J* = 8.7, 9.2 Hz, H-1′), 3.71 (d, 1H, *J* = 9.6 Hz, H-5′), 3.63 (s, 3H, OCH_3_), 3.54 (d, 2H, *J* = 4.1 Hz, indole-CH_2_), 3.31 (m, 1H, H-2′), 3.25 (m, 1H, H-4′), 3.18 (m, 1H, H-3′). ESI-MS: *m*/*z* [M+H]^+^: 365.1.

#### *N*,*N*-1,2-di-(1-deoxy-1-*N*-(2-(indol-3-yl)acetamide)-β-d-glucuronamide)ethane (**3d**)

This compound was prepared from **2d** (0.6 g, 1.7 mmol) and ethylenediamine (0.05 g, 0.84 mmol) using the same procedure as described for **3a**. The mixture was stirred under reflux for 1 week, the precipitate formed was filtered and washed with hot methanol (10 mL × 3), then dried under high vacuum, and **3d** (80 mg, 13.4% yield) as a brown solid was obtained. mp > 250 °C. ^1^H NMR (DMSO-*d*_6_, 600 MHz) δ: 10.86 (s, 1H, indol-NH), 8.63 (d, 1H, *J* = 9.2 Hz, sugar-NH), 8.08 (s, 1H), 7.56 (d, 1H, *J* = 7.8 Hz, H-4), 7.32 (d, 1H, *J* = 7.8 Hz, H-7), 7.19 (s, 1H, H-2), 7.05 (dd, 1H, *J* = 7.4, 7.3 Hz, H-5), 6.95 (dd, 1H, *J* = 7.7, 6.9 Hz, H-6), 5.13 (dd, 2H, *J* = 4.1, 4.6 Hz, OH), 5.00 (d, 1H, *J* = 5.5 Hz, OH), 4.76 (dd, 1H, *J* = 9.2, 8.7 Hz, H-1′), 3.54 (s, 2H, indol-CH_2_), 3.51 (d, 1H, *J* = 9.6 Hz, H-5′), 3.33 (m, 1H, H-2′), 3.18 (m, 2H, H-4′, H-3′), 3.09 (b, 2H, NCH_2_); ^13^C NMR (DMSO-*d*_6_, 150 MHz) δ: 171.3, 168.5, 136.0, 127.3, 123.8, 120.9, 118.9, 118.2, 111.2, 108.4, 79.9, 77.5, 77.1, 72.1, 71.2, 38.1, 32.6; ESI-MS: *m*/*z* [M+H]^+^: 725.2. HRMS(ESI): calcd. for C_34_H_41_N_6_O_12_^+^, 725.2782, found 725.2761.

## 4. Conclusions

DNA bis-intercalators has drawn considerable attention because of higher DNA-binding constants, slower dissociation rates and substantial sequence selectivity. We designed and synthesized a series of novel potential DNA bis-intercalators, in which two glucuronic acids were linked by ethylenediamine, and the glucuronic acid was coupled with various chromophores, including quinoline, acridine, indole and purine, at the C-1 position. The preliminary binding properties of these compounds to calf thymus DNA (CT-DNA) have been investigated by UV-absorption and fluorescence spectroscopy. The results indicated that all the target compounds can interact with CT-DNA, and the acridine derivative, **3b**, showed the highest key selection vector (KSV) value, which suggested that compound **3b** binds most strongly to CT-DNA and deserves further development.

## Supporting Information Available

^1^H NMR and ^13^C NMR spectra for new compounds are shown in supplementary material.

## Figures and Tables

**Table 1:** The key selection vector (KSV) value of compound **3a**–**3d**.

Entry	Compound 3a	Compound 3b	Compound 3c	Compound 3d
*ksv*	1.30 × 10^4^	1.54 × 10^4^	3.4 × 10^3^	1.04 × 10^4^
*R* (for 5 points)	0.998	0.982	0.995	0.998

## Supplementary Files

Supplementary File 1 Supporting Information (PDF, 269 KB)
