# Supplementary material for: Design, Synthesis and DNA Interaction Study of New Potential DNA Bis-Intercalators Based on Glucuronic Acid
Source: Int J Mol Sci. 2013 Aug 15;14(8):16851–65. doi: 10.3390/ijms140816851 (PMC3759939; doi:10.3390/ijms140816851)
Supplement: Supplementary File 1 — Supporting Information (PDF, 269 KB) [file ijms-14-16851-s001.pdf]

Supporting Information

^{1}H and ^{13}C NMR spectra of Compounds **3a**–**3d**.

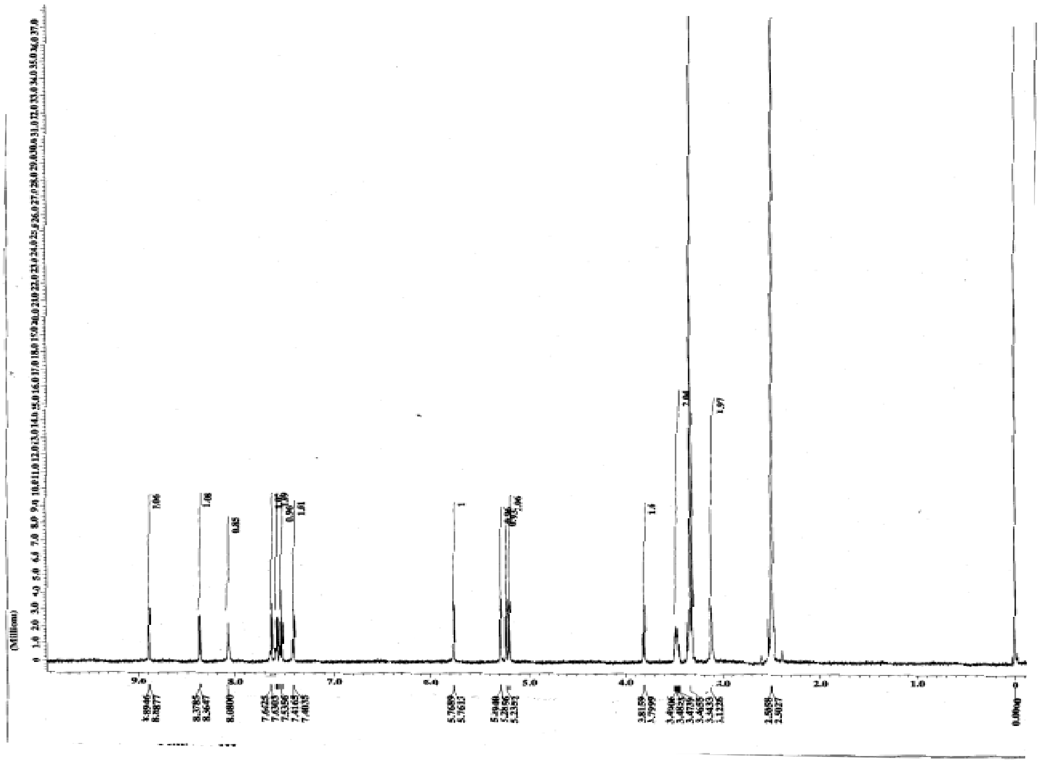

^{1}H NMR of compound **3a**.

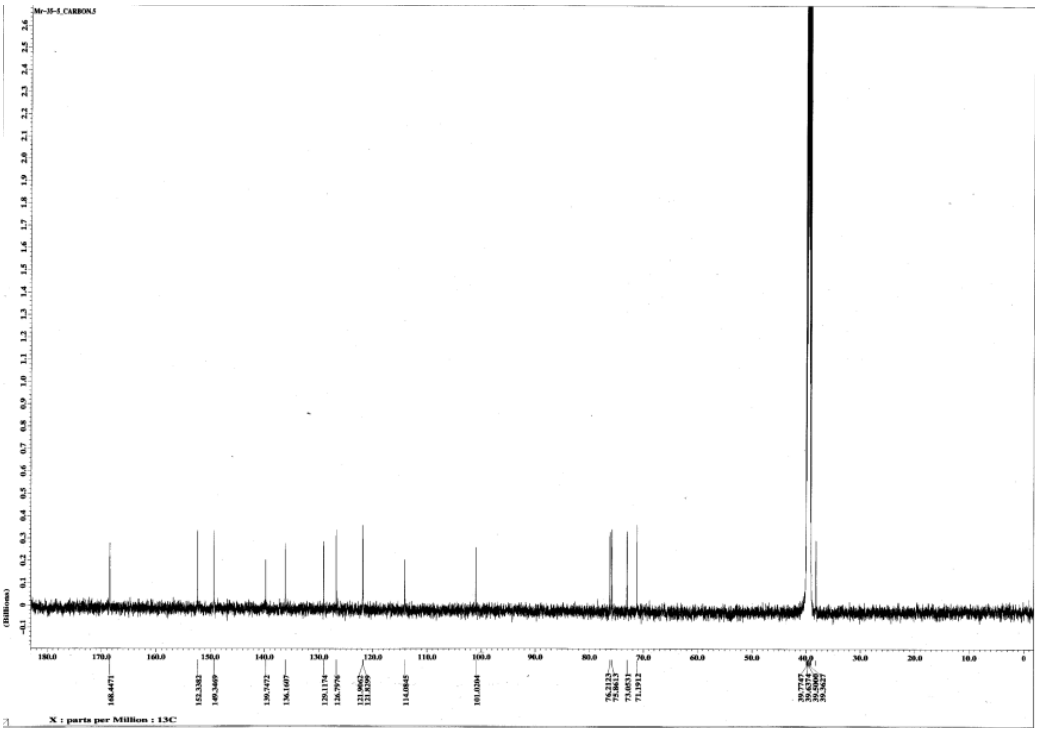

^{13}C NMR of compound **3a**.

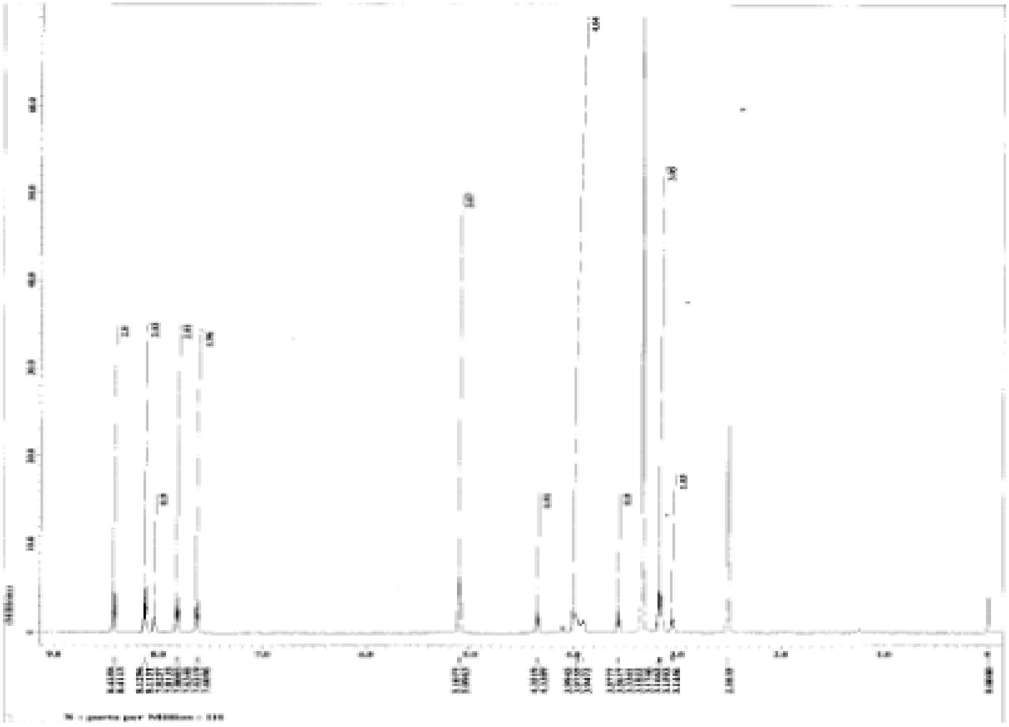

^{1}H NMR of compound **3b**.

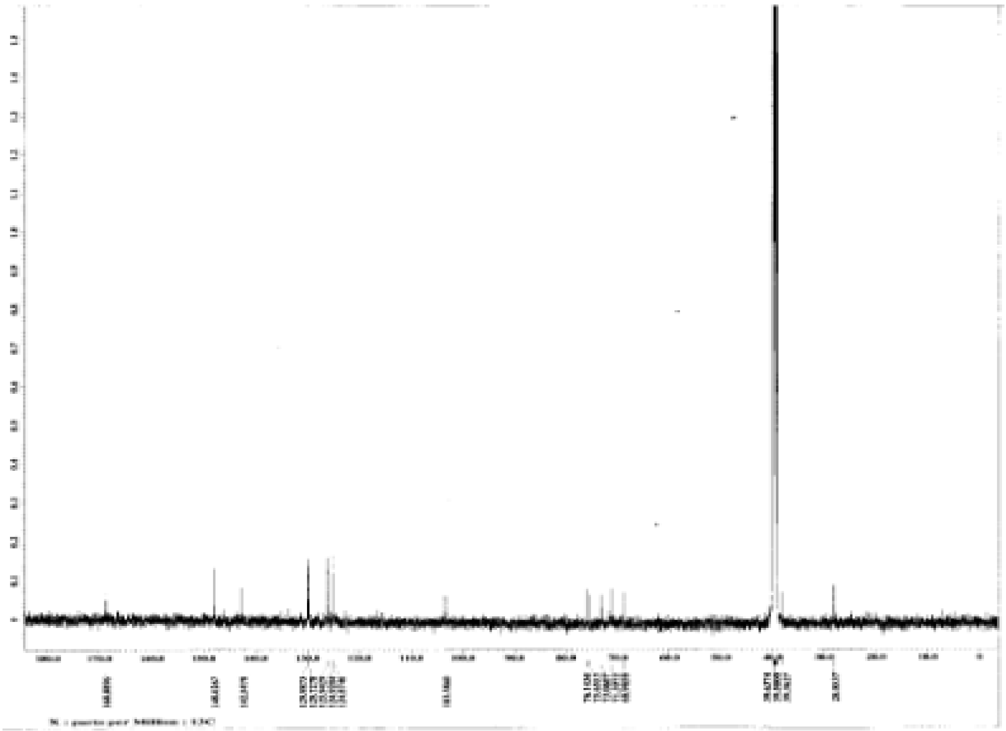

^{13}C NMR of compound **3b**.

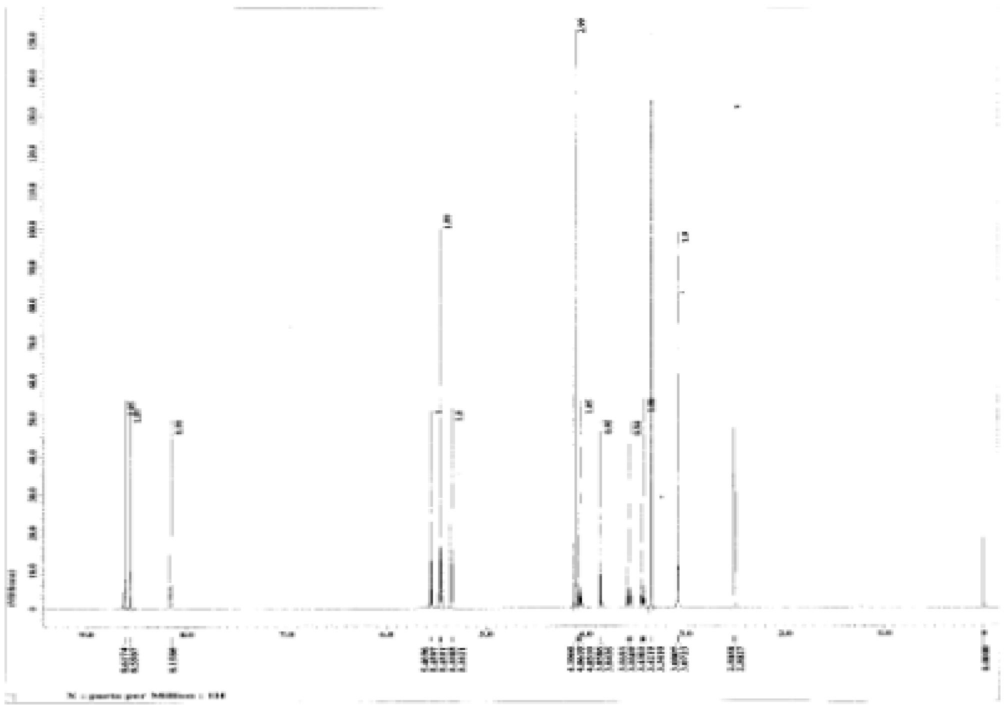

^{1}H NMR of compound **3c**.

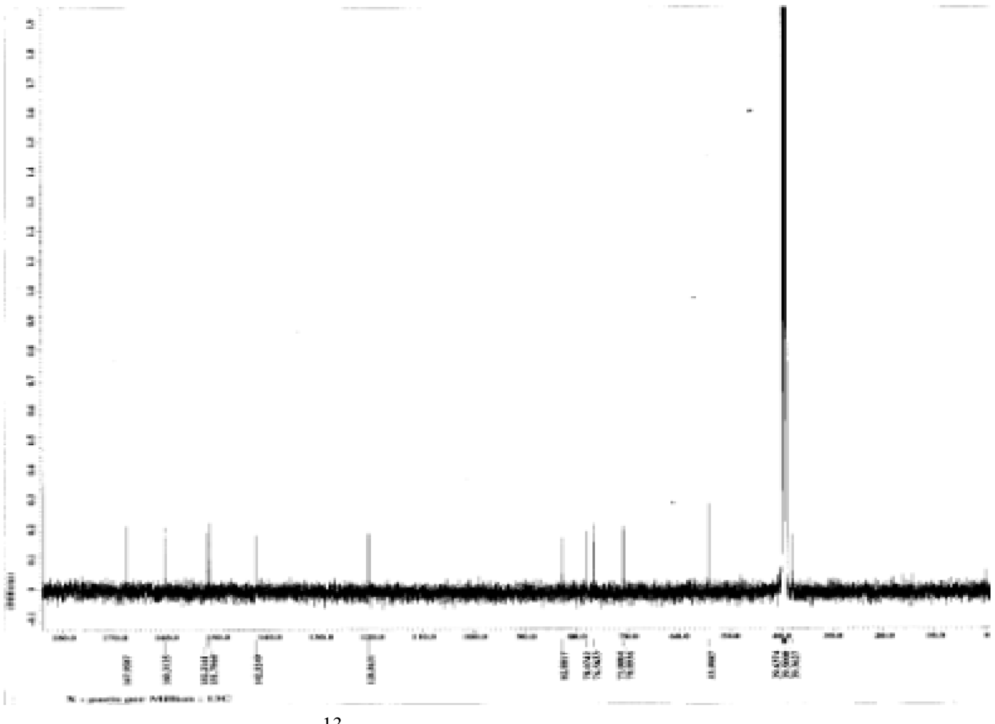

^{13}C NMR of compound **3c**.

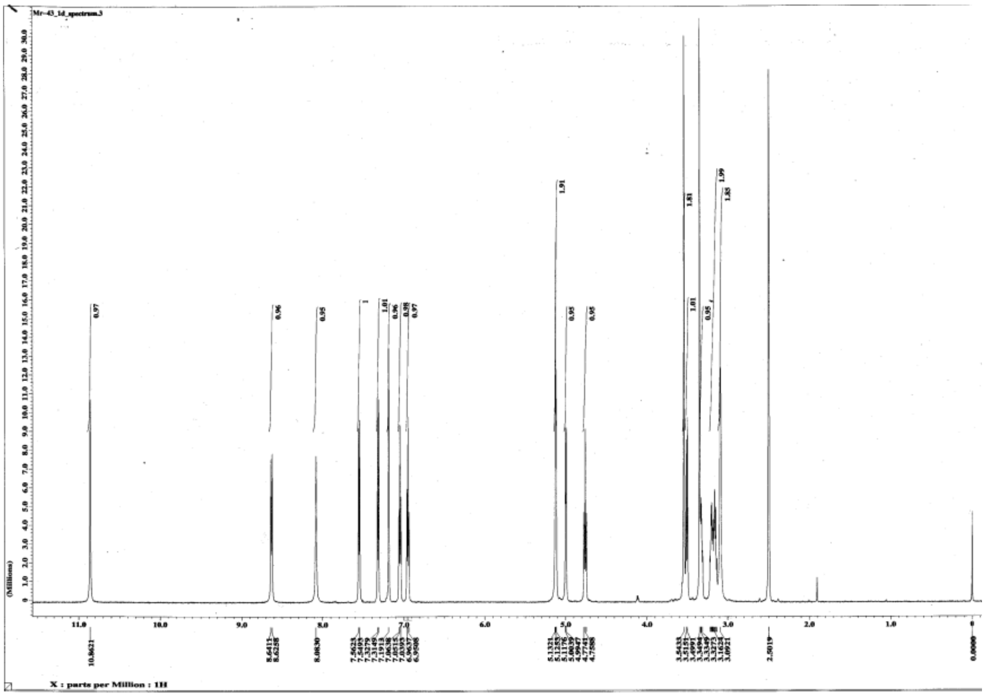

^{1}H NMR of compound **3d**.

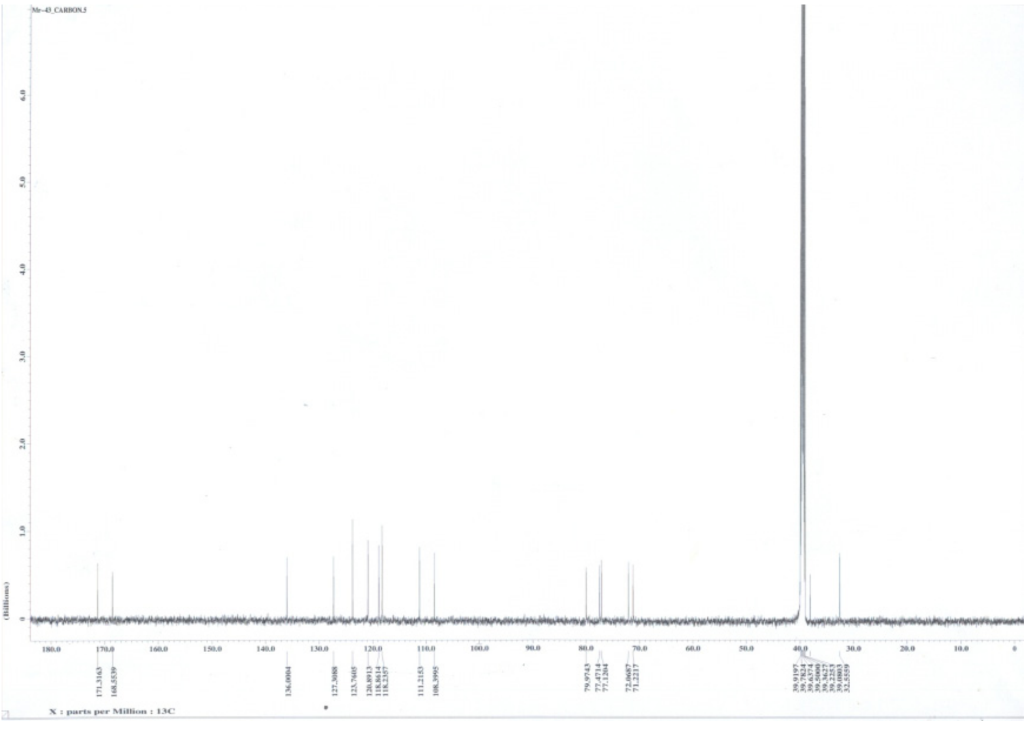

^{13}C NMR of compound **3d**.

© 2013 by the authors; licensee MDPI, Basel, Switzerland. This article is an open access article distributed under the terms and conditions of the Creative Commons Attribution license (http://creativecommons.org/licenses/by/3.0/).
